# Exploring the Holdase Activity of Supramolecular Chaperones with Amyloid-Forming Peptides and Insulin

**DOI:** 10.1021/acs.biomac.5c02408

**Published:** 2026-04-16

**Authors:** Elizabeth R. Piedmont, Hannah E. Distaffen, Lisbeth C. Crompton, Todd D. Krauss, Bradley L. Nilsson, Benjamin E. Partridge

**Affiliations:** † Department of Chemistry, 6927 University of Rochester, Rochester, New York 14627-0216, United States; ‡ Materials Science Program, University of Rochester, Rochester, New York 14627-0166, United States; § Institute of Optics, University of Rochester, Rochester, New York 14627-0186, United States

## Abstract

Protein folding is essential for maintaining cellular homeostasis. When proteins misfold, aggregation can occur, contributing to a variety of diseases. Amphiphilic naphthyl–benzyl ether dendrons were recently reported to mimic natural chaperone systems by reducing the extent of fibrillation of an amyloid beta (Aβ) peptide fragment. Herein we develop this system using a slower-aggregating mutant Aβ peptide and the essential therapeutic protein, insulin. We show that amphiphilic dendrons strictly mimic the holdase function of chaperones by preventing aggregation, rather than slowing aggregation or disaggregating preformed fibrils. We demonstrate that the activity of these molecules tolerates minor changes to their structure and translates from the model Aβ peptide to insulin without structural optimization. Moreover, second-generation dendron **2** effectively eliminates insulin aggregation for multiple days in the presence of chemical and mechanical stressors. These findings expand both our understanding and the potential therapeutic relevance of amphiphilic dendrons as an emerging approach to tackle protein aggregation.

## Graphical abstract

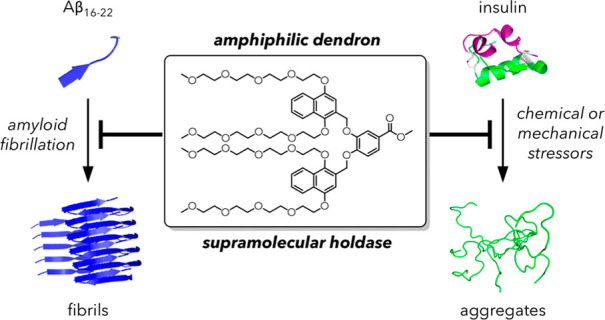

## Introduction

Proteins are the most diverse biomacromolecules^1^ in living systems and serve essential functions ranging from catalysis^2^ and transportation of cargo,^3^ to signaling transduction^4^ and cell replication.^5^ The information that encodes a protein’s function is stored within only 20 canonical amino acids, yet proteins achieve functional diversity via hierarchical folding into tertiary and quaternary structures that introduce complexity.^6^ Biological systems utilize chaperone proteins to assist the folding of other proteins into their native state (i.e., foldases^7,8^ ); in addition to protein folding, chaperones can stabilize proteins to prevent aggregation (i.e., holdases^9^) and even disaggregate protein fibrils (i.e., disaggregases^10^). Each type of chaperone is essential to maintain proteostasis, and when the integrity of this process is lost, protein aggregates can accumulate.^11^ Such aggregates are implicated in a variety of diseases, such as Alzheimer’s disease,^12^ Parkinson’s disease,^13^ and cataracts.^14,15^ Aggregation also contributes to the instability of therapeutic proteins, such as insulin and monoclonal antibodies, hindering the widespread use of biologics as drugs.^16–18^

To address the issue of protein aggregation, artificial chaperone systems have been developed to mimic the functions of their natural counterparts by leveraging electrostatic interactions or, more commonly, the hydrophobic effect.^19^ Hydrophobic systems have been developed that utilize both low molecular weight molecules (e.g., detergents)^20,21^ and high molecular weight polymers (e.g., poly­(ethylene glycol), PEG)^22,23^ to stabilize and/or refold proteins. However, current biomimetic approaches often interact too strongly with protein substrates, reducing protein recovery yields and limiting their application as therapeutics.^6,24^ Therefore, the development of adaptive, tunable, artificial systems remains a challenge.

We recently reported a series of amphiphilic dendrons as a new class of supramolecular holdase chaperones.^25^ Naphthyl–benzyl ether dendrons **1** and **2** (Figure 1) exhibit supramolecular assembly in water, forming sphere-like nanoparticles with low polydispersity. Addition of **1** or **2** to solutions of amyloid beta peptide fragment 16–22 (Aβ_16–22_), which is known to aggregate into amyloid fibrils in solution,^30,31^ reduced the extent of peptide fibrillation at substoichiometric quantities (30 μM dendron vs 200 μM peptide). Though our prior work introduced the potential of these molecules to mimic natural chaperone function, several questions about their mechanism and generality remained: (1) do amphiphilic dendrons inhibit peptide aggregation or break down assembled fibrils; (2) how tolerant is the activity of these dendrons to their molecular structure; and, (3) can these dendrons abrogate the aggregation of larger peptides or proteins? Herein we address these questions by exploring the activity of a library of amphiphilic dendrons against a mutant Aβ_16–22_ peptide with slower aggregation kinetics and by applying these amphiphiles to the therapeutically relevant protein, insulin. We show that the activity of naphthyl–benzyl ether dendrons proceeds via a holdase mechanism and is tolerant to minor structural changes to the dendron. Notably, these dendrons are also potent inhibitors of insulin aggregation for multiple days, induced by either a chemical denaturant or mechanical agitation.

**Figure 1:**
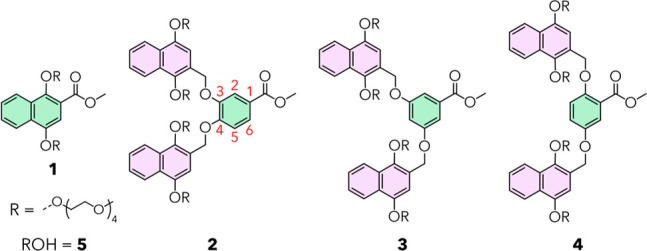
Structures of amphiphilic dendrons **1** to **4** and tetra­(ethylene glycol) monomethyl ether (**5**) investigated in this work. Green and pink indicate first- and second-generation branching units, respectively. Red numbering in **2** highlights the numerical position of the branching units.

## Experimental Section

### Synthesis

The synthesis of compounds **1** to **4** and of F19V peptide is described in the Supporting Information.

### Congo Red Peptide Binding Assays

CR binding assays were recorded on an Agilent Cary 3500 Multicell Peltier UV–vis spectrometer. Spectra were recorded with a scanning speed of 3000 nm/min, average time of 0.020 s, data interval of 1 nm, and spectral bandwidth of 2.00 nm. Solutions were measured at 25 °C in a capped quartz cuvette (Starna Cells) with a path length of 1 mm. *A*_540_/*A*_490_ values were calculated by dividing the absorbance value at 540 nm by the absorbance value at 490 nm. Solutions were prepared with spectrophotometric grade dimethyl sulfoxide (DMSO, Thermo Fisher Scientific) and phosphate-buffered saline (PBS) prepared using water purified to a resistivity of 18.2 MΩ·cm using a Milli-Q EQ 7008 water purification system (hereafter, “Milli-Q water”).

For time-course measurements of F19V aggregation, data were collected from 200 to 800 nm at 15 min intervals for the first 24 h, then at 24 h intervals for the remaining 7 days. Solutions of CR alone were prepared from 1× PBS stock solutions (500 μM) and diluted to reach a final concentration of 100 μM in 5% DMSO/1× PBS. To prepare samples containing F19V, glass vials were first charged with the appropriate volumes of 1× PBS solutions of CR (500 μM), dendron (100–500 μM), and/or **5** (Combi-Blocks, 300 μM). Additional 1× PBS was added to reach a volume of 285 μL. A stock solution of lyophilized F19V in DMSO (2 mM) was prepared and 15 μL of this solution was immediately added to each vial to reach a final F19V concentration of 100 μM and a total volume of 300 μL. The *A*_540_/*A*_490_ values calculated at *t* = 8 d were analyzed using a nested 1-way ANOVA test with Dunnett’s multiple comparisons test, comparing each sample pairwise with the data for CR + F19V.

**Figure 2:**
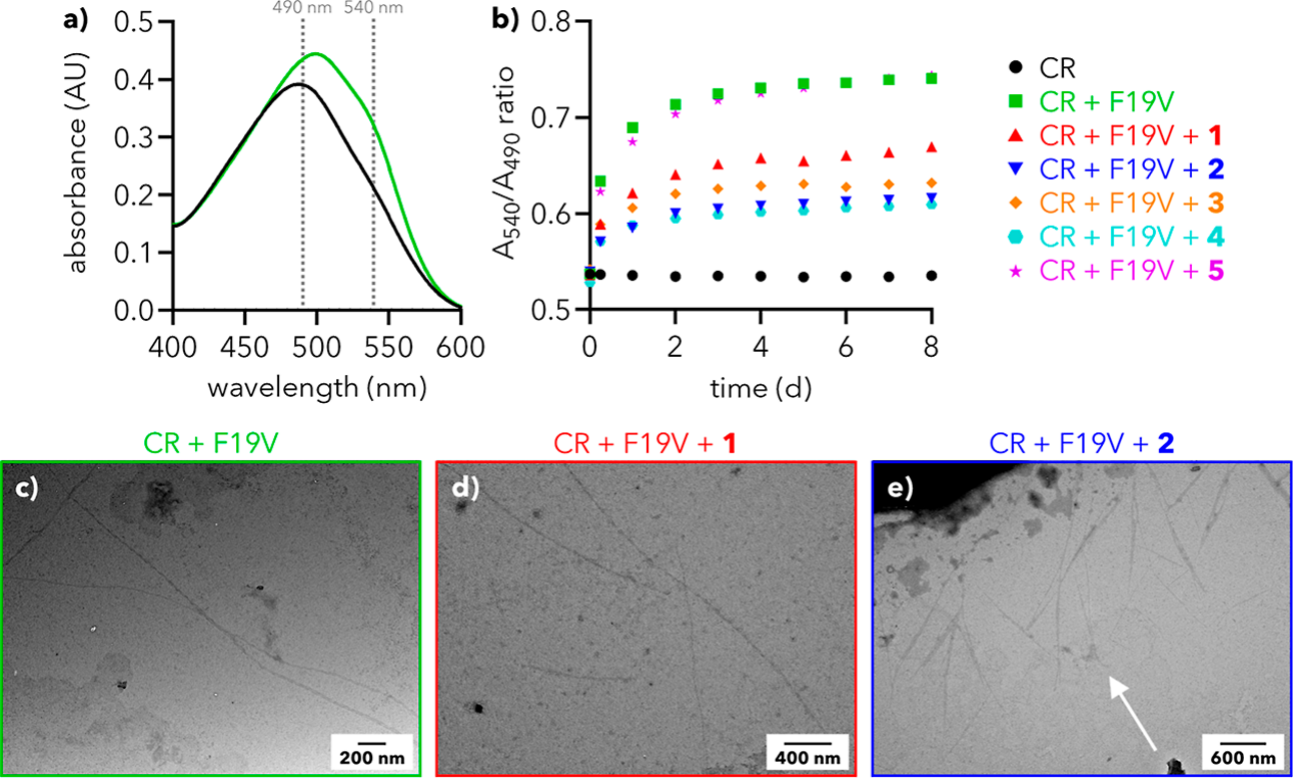
Congo red (CR) binding assays with F19V. (a) UV–vis spectra of CR (100 μM) alone [black] and with F19V (100 μM) [green] after 8 days at 25 °C in 5% DMSO/PBS. The characteristic shift in the λ_max_ of CR upon binding to fibrils can be seen. (b) *A*_540_/*A*_490_ values for solutions of CR alone (100 μM), CR and F19V (100 μM), and CR, F19V and either **1**, **2**, **3**, **4** (30 μM) or **5** (120 μM). Data represent the average of three replicates; error ranges are plotted in Figures S7 and S8. Control experiments of CR with dendrons are provided in Figure S9. (c–e) TEM images of CR and F19V mixtures after 8 days stained with NanoVan. The white arrow in (e) highlights shortened fibrils found in solutions of CR and F19V with **2**. Additional images in Figure S10.

For disaggregation assays , seven samples (300 μL solutions in 1 mm cuvettes) were prepared as described above for time-course measurements: one cuvette contained CR alone (100 μM) while the other six each contained a mixture of CR (100 μM) and F19V (100 μM). Absorbance data were collected for 8 d in the same manner as the time-course measurements described above. At day 8, samples were inverted three times to mix. Subsequently:

- To the cuvette containing CR alone, 45 μL of 5% DMSO/1× PBS was added (“CR”);
- To the cuvettes containing CR and F19V, 45 μL of 5% DMSO/1× PBS (“CR + F19V”) or 45 μL of a solution of compound **1**, **2**, **3**, **4**, or **5** (“CR + F19V + *x*”) in 5% DMSO/1× PBS was added.
- Samples were inverted three times to mix, and data were collected from 200 to 800 nm at 15 min intervals for 24 h at 25 °C.

**Figure 3:**
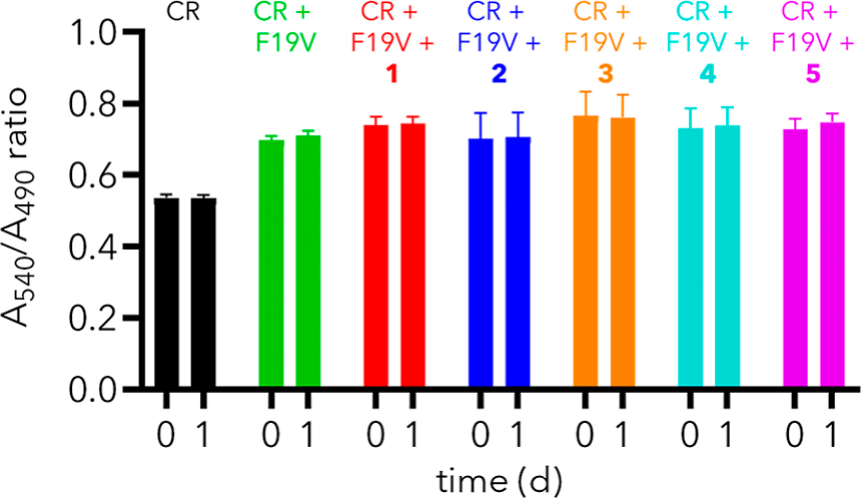
Disaggregation study with mature F19V fibrils. Mature F19V fibrils were prepared by incubating solutions of CR (100 μM) and F19V (100 μM) at 25 °C for 8 days. Subsequently, solutions of **1**, **2**, **3**, **4**, or **5** in 5% DMSO/PBS were added and the absorbance measured (*t* = 0 d). Mixtures were incubated for 24 h at 25 °C and their absorbance after 24 h is shown (*t* = 1 d). Error bars indicate mean ± SD (*n* = 3).

### Insulin Aggregation Studies

Turbidity assay data were recorded on a Biotek PowerWave XS plate reader. Measurements were taken at 25 °C with continuous shaking at the instrument’s preset “slow” rate for all experiments, except the high agitation experiment which was continuously shaken at the instrument’s preset “fast” rate. All solutions for turbidity assays were prepared using 1× PBS that was filtered with a 0.2 μm syringe filter immediately prior to use.

**Figure 4:**
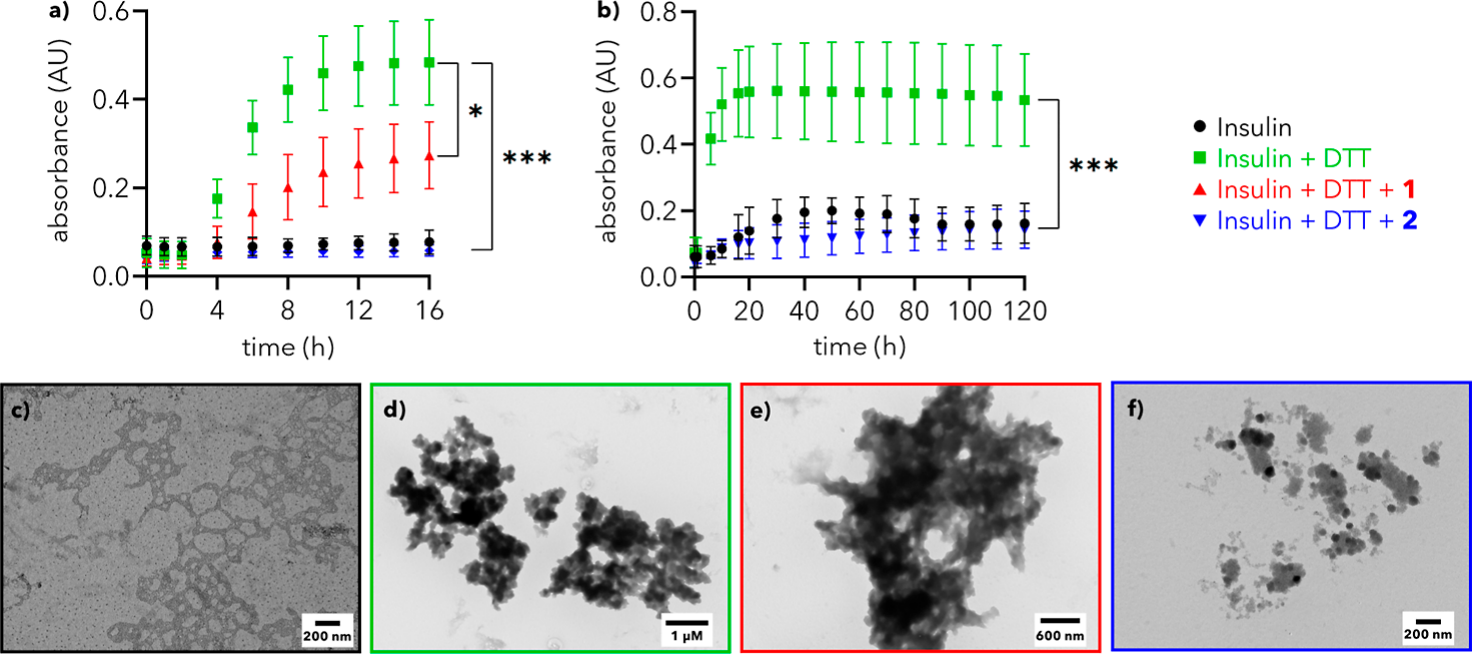
Insulin turbidity assay in reducing conditions. (a) 16 h insulin turbidity assay. *A*_450_ values of solutions of insulin (0.4 mg/mL) alone, with DTT (100 μM), and with DTT and either **1** or **2** (250 μM) at 25 °C. Error bars indicate mean ± SD (*n* = 3). (b) 5 Day insulin turbidity assay. *A*_450_ values of solutions of insulin (0.4 mg/mL) alone, with DTT (100 μM), and with DTT and **2** (250 μM) at 25 °C. Error bars indicate mean ± SD (*n* = 4). * denotes *p* ≤ 0.05; ** denotes *p* ≤ 0.01; *** denotes *p* ≤ 0.001. (c–f) TEM images of solutions from (a) after 16 h stained with NanoVan.

**Figure 5:**
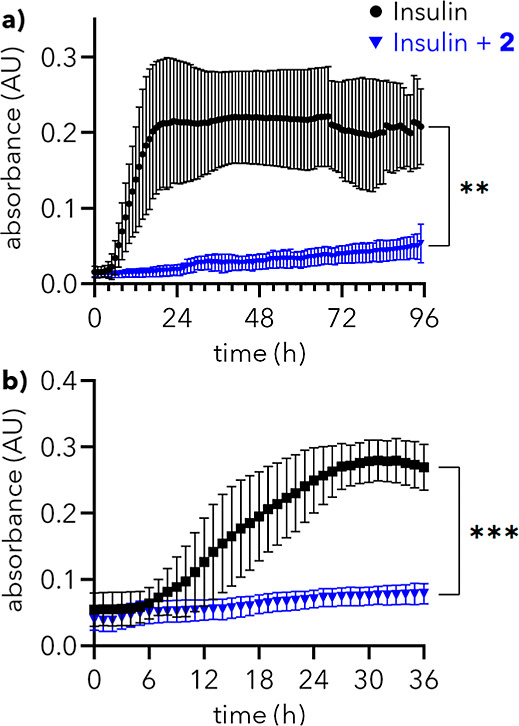
Native insulin turbidity assays. (a) Extended duration assay. *A*_450_ values of solutions of insulin (0.4 mg/mL) alone and with **2** (250 μM) at 25 °C for 95 h. (b) Aggressive agitation assay. *A*_450_ values of solutions of insulin (0.4 mg/mL) alone and with **2** (250 μM) agitated with fast shaking at 25 °C for 36 h. In (a,b), error bars indicate mean ± SD (*n* = 3). * denotes *p* ≤ 0.05; ** denotes *p* ≤ 0.01; *** denotes *p* ≤ 0.001.

### DTT-Induced Insulin Aggregation

A stock solution of dithiothreitol (DTT, Research Products International, 1200 μM) was prepared in a 10 mL volumetric flask, and 2.5 mL of this stock solution were transferred to a clean 10 mL volumetric flask. Separately, 7.53 μmol of dendron **1** or **2** were weighed in a glass vial and dissolved in ∼3 mL of 1× PBS. The entire dendron solution was transferred, with rinsings, into the volumetric flask containing 2.5 mL of DTT solution. The volume was made up to 10 mL with 1× PBS to give a stock solution containing dendron (753 μM) and DTT (300 μM). A solution containing only DTT (300 μM) was also prepared by serial dilution of the 1200 μM stock using a 10 mL volumetric flask. For concentration-dependent experiments, a separate dendron-DTT stock solution was prepared for each dendron concentration to keep the volume added to the plate consistent. For 50, 100, 150, 200, and 250 μM concentrations of dendron **2** were weighed out 1.51, 3.01, 4.53, 6.02, and 7.53 μmol of dendron **2**, respectively.

### Native Insulin Aggregation

Stock solutions of dendron **2** were prepared as described above, except that the addition of 2.5 mL of DTT stock solution was omitted. The volume was made up to 10 mL with 1× PBS to give a stock solution containing dendron only (753 μM).

All solutions containing dendron (with or without DTT) were allowed to sit at room temperature for 15 min to ensure complete dissolution. After 15 min, insulin (1.2 mg, MP Biomedicals) was added to 2 mL of 1× PBS in a glass vial and gently agitated on a tube rocker for 15 min to ensure dissolution. Solutions were added directly to a 384-well plate (Invitrogen) in the following order:

- insulin (33.4 μL; final concentration = 0.4 mg/mL);
- 1× PBS (as needed to bring the final total well volume to 50 μL);
- solution containing DTT and/or dendron (16.6 μL; final concentration = 0 or 100 μM DTT and 0–250 μM dendron, as applicable).

The 384-well plate was sealed with a plastic lid that was further secured with electrical tape. The plate was shaken within the plate reader for 10 s prior to the start of data collection to ensure thorough mixing, after which the absorbance at 450 nm was measured at intervals of 10 min for 16 h experiments, or at intervals of 1 h for 36 h, 4 d, and 5 d experiments. *A*_450_ values measured at *t* = 16 h, 36 h, 4 d, or 5 d, respectively, were analyzed using a nested 1-way ANOVA test with Tukey’s multiple comparisons test for all possible pairwise comparisons.

### Transmission Electron Microscopy

TEM measurements were performed on a Hitachi 7650 TEM working at 80 kV with an attached 11-megapixel Gatan Erlangshen digital camera for image capture. Images were recorded using Digital Micrograph software. TEM images from both CR assays and insulin aggregation assays were recorded on aliquots taken directly from the cuvette at 8 days or directly from the well plate at 16 h, respectively. Samples for imaging were prepared on 200 mesh silicon/Formvar grids (Ted Pella) by sequential treatment with the following solutions, wicking away excess solution with filter paper after each step: 10 μL of the solution for analysis, incubated for 2 min; 10 μL of Milli-Q water to remove buffer salts, immediately wicked away; 10 μL of NanoVan stain (Nanoprobe, diluted 1:1 with Milli-Q water), incubated for 2 min; and 10 μL of Milli-Q water to remove excess stain, immediately wicked away. Grids were allowed to dry under ambient conditions for at least 10 min prior to imaging.

### Atomic Force Microscopy

AFM measurements were performed at ambient temperature on an Asylum MFP-3D-BIO AFM (Oxford Instruments) using the Asylum Research version 12 software. AFM was operated in tapping mode with 240AC-NA cantilevers (NanoAndMore) with spring constants of ∼2 N/m at their resonant frequencies of ∼70 kHz. Image analysis was performed with Igor Pro 6.3.7.2. Dendron stock solutions (100 or 300 μM) were prepared and diluted to reach a final concentration of 30 μM in Milli-Q water. Solutions were then spin-coated (50 μL) at 3000 rpm for 60 s onto grade V-4 freshly cleaved mica substrate (Ted Pella).

## Results and Discussion

Inspired by natural holdase chaperones that utilize hydrophobic surfaces to interact with exposed nonpolar residues on misfolded proteins,^9,26^ previously reported dendrons **1** and **2** were synthesized with an extended hydrophobic core based on naphthyl and benzyl ethers (Figure 1).^25^ Naphthyl moieties were selected due to their propensity to induce assembly and because they are a well-tolerated molecular motif found in numerous drugs including naproxen and tolnaftate.^27^ At the periphery of each molecule, tetra­(ethylene glycol) chains were installed to ensure water solubility while favoring supramolecular assembly in aqueous media. The generation number of the dendron—i.e., how many levels of branching are present in each dendron—was varied to modulate their assembly properties. Second-generation dendron **2**, with both naphthyl and benzyl ether branching units, has more nonpolar aromatic groups than first-generation dendron **1**, decreasing its water solubility but increasing its hydrophobicity and propensity to assemble.

To explore how more subtle changes in molecular structure affect chaperone activity, constitutionally isomeric second-generation dendrons **3** and **4** were synthesized (Figure 1). The synthesis of dendrons **3** and **4** follows a similar synthetic route to that of **2** and is described in the Supporting Information (Scheme S1 and Figures S1–S4). Dendrons **3** and **4** differ from **2** only in the attachment pattern of naphthyl ethers to the benzyl core: the naphthyl branching units are attached at the 3,4-, 3,5- and 2,5-positions for dendrons **2**, **3**, and **4**, respectively. Constitutional isomers of benzyl ether dendrons are known to exhibit structure-dependent assembly,^28,29^ and we hypothesized that changing the attachment positions at the benzyl ether branching units in second-generation dendrons may lead to a difference in chaperone activity. Atomic force microscopy (AFM) reveals that changes in the molecular structure of second-generation dendrons do affect aqueous supramolecular assembly. In solution, dendron **2** shows particles with low dispersity and apparent diameters of 10 ± 2 nm (mean ± SD, Figure S5a). In contrast, dendrons **3** and **4** form larger, ill-defined assemblies with diameters of 38 ± 18 nm and 29 ± 18 nm, respectively (Figure S5b–d). Both dendrons **3** and **4** are water-soluble, albeit substantially less so than second-generation dendron **2** (∼100 μM for **3** and **4** vs ∼1000 μM for **2**), which could contribute to the formation of particles with increased polydispersity in solution.

The chaperone activity of dendrons **1** and **2** was previously assessed by monitoring the extent of fibrillation of wild-type Aβ_16–22_.^25,30^ Under our experimental conditions (200 μM peptide in water), peptide aggregation was completed within 30 min and thus we were unable to determine whether dendrons were slowing peptide aggregation, preventing peptide aggregation, or disaggregating already-assembled peptide fibrils. To distinguish between these three mechanisms, a slower-aggregating mutant of Aβ_16–22_ was utilized to ensure that dendrons could be added prior to fibril formation. Specifically, mutating the residue at position 19 from phenylalanine to valine (F19V) has been shown to increase the residual monomer concentration after aggregation is completed to 58 ± 3 μM (vs 33 ± 3 μM for the wild-type peptide) and reduce its aggregation rate substantially.^31^ Consequently, F19V was selected as a model substrate and aggregation studies were conducted over 8 days to ensure complete fibrillation.

Amyloid fibrillation was monitored using the colorimetric probe, congo red (CR), due to its characteristic bathochromic shift in maximum absorbance (λ_max_) from 490 to 540 nm upon binding to amyloid fibrils.^32,33^ Due to the spectral overlap between free CR (λ_max_ = 490 nm) and peptide-bound CR (λ_max_ = 540 nm), it is convenient to quantify the extent of fibrillation by analyzing the ratio of the absorbance at these two values, i.e. *A*_540_/*A*_490_, such that an increase in fibrillation leads to an increase in peptide-bound CR and a concomitant increase in the *A*_540_/*A*_490_ ratio.

At 8 days, a solution of CR (100 μM) in 5% dimethyl sulfoxide (DMSO)/phosphate-buffered saline (PBS, pH 7.4) shows its characteristic λ_max_ of 490 nm with a *A*_540_/*A*_490_ ratio of 0.54 (Figure 2a, black). Incubation of F19V (100 μM, Figure S6) together with CR (100 μM) over the same time period produces a UV spectrum with a clear additional feature at 540 nm, with an associated *A*_540_/*A*_490_ ratio of 0.74 (Figure 2a, green). Time-course monitoring shows that this ratio increases monotonically over the observation period, increasing rapidly within the first 48 h and reaching a plateau by 8 days (Figure 2b, green).

The addition of dendron (30 μM) at the start of the 8 day incubation period reduced the overall increase in *A*_540_/*A*_490_ ratio, indicative of a reduction in the extent of peptide fibrillation (Figure 2b). First-generation dendron **1** exhibited a modest reduction in overall fibrillation (*A*_540_/*A*_490_ = 0.67 at 8 days vs 0.74 with no dendron; *p* = 0.47) that was unfortunately highly variable between replicates; data with error ranges are shown in Figures S7 and S8. Transmission electron microscopy (TEM) images of mixtures of F19V, CR, and **1** primarily show long, extended fibrils similar to F19V without dendron (Figure 2c,d). Second-generation dendrons **2** to **4** also show reduced *A*_540_/*A*_490_ ratios: 0.62 for **2** (*p* = 0.11), 0.63 for **3** (*p* = 0.04), and 0.61 for **4** (*p* = 0.08). The similarity of the data for mixtures of F19V and dendrons **2** to **4** suggests that minor changes to the hydrophobic core of these dendrons do not impact their chaperone activity. It is noteworthy that the *A*_540_/*A*_490_ values observed for mixtures of F19V and dendron all reach a plateau within 8 days, and that the plateau value remains lower than that observed for solutions without dendron, signifying that dendrons are not simply slowing aggregation but are instead preventing it. This is supported by the observation of both long and short fibrils in mixtures of F19V and **2** (Figure 2e, additional images in Figure S10).

Given the reported ability of PEGs to stabilize proteins from aggregating,^22,34,35^ control experiments were conducted with tetra­(ethylene glycol) monomethyl ether (**5**), the hydrophilic component of dendrons **1** to **4** (structure in Figure 1). To account for the four ethylene glycol chains per second-generation dendron, F19V and CR were incubated with 120 μM of **5** (compared to 30 μM of **2**, **3**, or **4**). Essentially no difference was observed between samples of F19V incubated with or without **5** (compare green and pink data in Figure 2b), indicating that the ethylene glycol chains alone are not sufficient to reduce fibrillation. This could suggest that the hydrophobic core provided by the naphthyl-benzyl ether dendrons is required for peptide stabilization. Alternatively, the covalent attachment of ethylene glycol chains to the dendron may reduce their conformational flexibility or increase their effective local concentration, thereby reducing the entropic barrier to associating with, and preventing the aggregation of, proteins.

The observation that samples of F19V with dendron reached a plateau with a lower *A*_540_/*A*_490_ value than that of F19V alone signifies that dendrons prevent, rather than merely slow, the aggregation of F19V peptides; that is, they are exhibiting holdase activity. To determine whether dendrons also exhibit disaggregase activity, solutions of CR and F19V were incubated at 25 °C over 8 days to form mature F19V fibrils. Subsequently, these fibrils were challenged with dendron (**1** to **4**) or control glycol **5** and their UV absorbance was monitored over 24 h (Figure 3). Addition of **1**, **2**, **3**, **4**, or **5** resulted in no change in *A*_540_/*A*_490_ ratio after 24 h, indicating that the fibrils present immediately prior to dendron addition were not disrupted and, therefore, that dendrons do not disaggregate mature fibrils. Hence these studies demonstrate that amphiphilic dendrons function strictly to prevent aggregation of amyloid peptides, mimicking the functions of a holdase chaperone. Moreover, these studies highlight that second-generation dendrons are tolerant to minor changes to their molecular structure; therefore, we focused on dendrons **1** and **2** for subsequent studies due to their greater aqueous solubility than **3** and **4**.

With a better understanding of how dendrons reduce fibrillation in a model peptide system, we were interested to see whether these molecules would retain efficacy with substrates larger than the seven-residue F19V Aβ_16–22_ fragment. To assess this, insulin was chosen as a model protein system. Insulin (51 amino acids, *M*_W_ = 5.8 kDa) is an essential therapeutic drug in the treatment of diabetes, listed on the WHO Model List of Essential Medicines,^36^ but is prone to aggregate at elevated temperatures,^37^ when subjected to mechanical agitation,^38,39^ or in the presence of chemical denaturants.^40–42^ This high propensity for insulin to aggregate reduces its shelf life, necessitates cumbersome cold-chain storage,^43^ and makes treatment less accessible for patients.^42,44^ Therefore, developing ways to stabilize insulin through improved formulation has become increasingly important. Small molecules,^45^ polycyclic aromatic compounds,^46,47^ peptides,^48,49^ supramolecular hosts,^50,51^ and polymers^52–54^ have been shown to stabilize insulin at various points along its aggregation pathway. However, no single strategy has yet proven suitable to maintain insulin stability throughout the entirety of its production, transportation, storage, and injection. Therefore, the development of molecules that stabilize insulin is still a necessity.

To induce aggregation reproducibly, monomeric insulin (Figure S11) was treated with the chemical denaturant dithiothreitol (DTT). Native insulin contains two subunits, chain A (21 aa, 2.4 kDa) and chain B (30 aa, 3.4 kDa), that are linked together via two disulfide bridges. The presence of reducing agents such as DTT reduces these disulfide bridges, thereby cleaving the insulin monomer and leading to protein aggregation.^41,55,56^ This aggregation can be monitored using standard turbidity assays, in which aggregates in solution lead to increased light scattering and an associated increase in absorbance above 400 nm.^41,55,57^ The change in absorbance over time correlates to the extent of aggregation.

A solution of insulin (0.4 mg/mL in PBS) incubated at 25 °C for 16 h shows little to no aggregation, as determined by monitoring the absorbance at 450 nm (Figure 4a, black). Control experiments with insulin and dendron **1** or **2** also show little aggregation (Figure S12a). As expected, incubation with DTT (100 μM) shows a clear increase in absorbance to 0.48 ± 0.10, signifying aggregation (Figure 4a, green). In contrast, a solution of insulin and DTT with second-generation dendron **2** (250 μM) exhibits essentially no increase in absorbance over 16 h (*A*_450_ = 0.06 ± 0.01), indicating that dendron **2** completely prevents the aggregation of insulin in the presence of DTT (Figure 4a, blue; *p* = 0.0001). A dendron concentration of 250 μM was chosen to ensure effectiveness in extended-duration turbidity assays (see Figures S13 and 5, below). Notably, first-generation dendron **1** (250 μM) also reduces the extent of aggregation (Figure 4a, red; *A*_450_ = 0.27 ± 0.08; *p* = 0.02) albeit less effectively than dendron **2**. This difference in stabilization by dendrons **1** and **2** highlights that large structural changes (i.e., first vs second generation) influence chaperone activity toward proteins and suggests that the extended hydrophobic core is beneficial for stabilization.^25^

At the end of the turbidity assay, samples were visualized by TEM to probe the extent of aggregation. Solutions of insulin alone show no apparent aggregates (Figures 4c and S14a,e) whereas insulin incubated with DTT exhibits large amorphous structures consistent with aggregation (Figures 4d and S14b,f).^41^ Mixtures of insulin, DTT, and **1** maintain the presence of these large aggregates (Figures 4e and S14c,g). Conversely, no large aggregates are visible in mixtures of insulin, DTT, and dendron **2** (Figures 4f and S14d,h), supporting the conclusion from turbidity assays (Figure 4a) that **2** reduces the extent of insulin aggregation in the presence of DTT.

Encouraged by the potent holdase activity of dendron **2** over 16 h, we questioned whether this efficacy would be maintained over longer time periods. Accordingly, turbidity assays were conducted over the course of 5 days. Solutions of insulin with DTT show a substantial increase in absorbance within the first 20 h, reaching a plateau *A*_450_ value that is stable for the remainder of the 5 day period (*A*_450_ at 5 *d* = 0.52; Figure 4b, green). Gratifyingly, incubation with **2** does not lead to an increase in absorbance and effectively eliminates insulin aggregation compared to the sample without dendron (Figure 4b, blue; *p* = 0.0002). Therefore, dendron **2** maintains the stability of cleaved insulin fragments for multiple days and suggests that **2** can exert holdase activity over an extended period.

Nuclear magnetic spectroscopy (NMR) and matrix-assisted laser desorption-ionization time-of-flight (MALDI-TOF) mass spectrometry were used to determine whether dendrons **1** and **2** inhibit insulin aggregation by preventing its cleavage by DTT or by stabilizing cleaved insulin fragments. ^1^H NMR spectra of DTT with dendron **1** or **2** show no change over 16 h (Figure S15), suggesting that dendrons do not serve as sacrificial oxidants for DTT. This is consistent with the lack of any change in UV absorbance for mixtures of dendron and DTT over 16 h (Figure S12b). MALDI-TOF spectra recorded for samples taken after the turbidity assay show that insulin is cleaved by DTT even in the presence of dendrons (Figure S16). In insulin alone, a peak corresponding to monomeric protein is observed at 5.8 kDa; this peak is absent for solutions of insulin and DTT and instead only a peak at 2.3 kDa is present, which is associated with insulin chain A. These data suggest that dendrons do not physically or chemically protect insulin from being cleaved by DTT and, therefore, dendrons likely stabilize the cleaved A- or B-chains against aggregation. Unfortunately, attempts to directly observe the formation of a complex between **2** and insulin using diffusion-ordered spectroscopy (DOSY) NMR were unsuccessful.

While DTT provides convenient and reproducible insulin aggregation through the cleavage of intrachain disulfide bonds, it does not accurately represent the aggregation pathway of native insulin. Native insulin aggregation is especially relevant to therapeutic applications of insulin as it is commonly stored in wearable point-of-care devices that must be changed every 48 to 72 h to maintain efficacy.^58–61^ Native insulin typically exists as a Zn^2+^-bridged hexamer that dissociates upon receptor binding to generate the active, monomeric form.^42,62^ When exposed to denaturing conditions, such as elevated temperature, agitation, changes in pH, and interactions with hydrophobic surfaces, monomeric insulin can partially unfold, leaving it susceptible to aggregation.^42^

Therefore, to better assess native insulin aggregation, insulin turbidity assays were conducted without the addition of chemical denaturants. Over 4 days, solutions of monomeric insulin (0.4 mg/mL) show mild, variable aggregation (Figure 5a, black). In contrast, the presence of **2** (250 μM) results in minimal change in absorbance over 95 h (Figure 5a, blue; *p* = 0.0018 at 95 h), indicating that **2** prevents the aggregation of native insulin over multiple days. Finally, to mimic the agitative environment of point-of-care devices caused by natural daily activity,^38^ solutions of insulin (0.4 mg/mL) at 25 °C were subjected to fast agitation over 36 h. While insulin alone shows substantial aggregation (Figure 5b, black), solutions of insulin with **2** (250 μM) exhibit minimal increase in absorbance, indicative of a near-complete abrogation of insulin aggregation (Figure 5b, blue; *p* = 0.0004 at 36 h). Taken together, these results evidence that dendron **2** can prevent the aggregation of native insulin over extended periods under conditions representative of point-of-care device storage.

## Conclusions

This work shows that amphiphilic dendrons based on naphthyl and benzyl ethers are effective mimics of natural holdase chaperones for small peptides and the therapeutically relevant protein, insulin. We find that these dendrons stabilize peptides with respect to aggregation by functioning purely as holdases rather than by breaking down preassembled fibrils. While substantial changes to the structure of the dendron (compare first-generation **1** vs second-generation **2**–**4**) lead to a significant difference in holdase efficacy, minor structural changes between second-generation dendrons are functionally well tolerated despite affecting their supramolecular assembly. The second-generation dendron with highest water solubility, dendron **2**, is shown to be an effective holdase for insulin over extended periods of time in the presence of the harsh chemical denaturant DTT (5 days) or when subjected to moderate (4 days) or aggressive (36 h) mechanical agitation. Notably, dendron **2** was able to exhibit holdase function against two distinct protein substrates without structural modification, obviating the need for tedious molecular optimization often inherent to small molecule approaches. Compared to polymeric stabilizing agents that exhibit optimal function at high concentrations,^52^ dendron **2** is effective as a minority component in solution (0.3 mg/mL **2** vs 0.4 mg/mL insulin in Figures 4 and 5). These results highlight the potential of amphiphilic dendrons as a promising class of protein aggregation inhibitors.

## Supplementary Material



## Data Availability

Raw and processed data used in this article are available at https://doi.org/10.60593/ur.d.30581678.

## Notes

Synthetic procedures and characterization of amphiphilic dendrons **1** to **4**; synthetic procedures and characterization of F19V; plots of individual replicates and control experiments for CR peptide binding assays; AFM images of dendron **2**, **3**, and **4**; supporting SEC, NMR, and MALDI-TOF data for insulin aggregation assays; additional TEM images for CR peptide binding assays and insulin aggregation assays; additional references (PDF)
