# Supplementary material for: Exploring the Holdase Activity of Supramolecular Chaperones with Amyloid-Forming Peptides and Insulin
Source: Biomacromolecules. 2026 Apr 16;27(5):3109–16. doi: 10.1021/acs.biomac.5c02408 (PMC13169323; doi:10.1021/acs.biomac.5c02408)
Supplement: Supplementary file 1 [file bm5c02408_si_001.pdf]

Supporting Information for

Exploring the Holdase Activity of Supramolecular Chaperones with Amyloid-Forming Peptides and Insulin

Elizabeth R. Piedmont,[1] Hannah E. Distaffen,[1] Lisbeth C. Crompton,[1] Todd D. Krauss,[1,2,3] Bradley L. Nilsson,[1,2] and Benjamin E. Partridge[1,2]*

[1]*Department of Chemistry, University of Rochester, Rochester, NY 14627-0216, United States.*
[2]*Materials Science Program, University of Rochester, Rochester, NY 14627-0166, United States.*
[3]*Institute of Optics, University of Rochester, Rochester, NY 14627-0186, United States.*

*Email: benjamin.partridge@rochester.edu

Table of Contents

1. Synthesis and Characterization of Amphiphilic Dendrons **1** to **4** S2
1.1. Materials and Methods S2
1.2. Experimental Procedures S3
Scheme S1 .... S3
Figure S1 .... S5
Figure S2 .... S6
Figure S3 .... S7
Figure S4 .... S8
2. Atomic Force Microscopy S9
Figure S5 .... S9
3. Synthesis and Characterization of F19V Peptide S10
Figure S6 .... S10
4. Supporting Data for Congo Red Peptide Binding Assays S11
4.1. Data for Individual Replicates S11
Figure S7 .... S11
Figure S8 .... S11
4.2. Control Experiments with Congo Red and Dendron S12
Figure S9 .... S12
4.3. Additional TEM Images S12
Figure S10 .... S12
5. Supporting Data for Insulin Aggregation Assays S13
5.1. Determination of Insulin Oligomerization State by SEC S13
Figure S11 .... S13
5.2. Control Experiments with Insulin or DTT and Dendrons S13
Figure S12 .... S13
5.3. Concentration-Dependent Aggregation Inhibition S14
Figure S13 .... S14
5.4. Additional TEM Images S15
Figure S14 .... S15
5.5. NMR Study of Dendron Stability with DTT S16
Figure S15 .... S16
5.6. MALDI-TOF MS Study of Insulin Cleavage with DTT S17
Figure S16 .... S17
6. References for the Supporting Information S18

1. Synthesis and Characterization of Amphiphilic Dendrons 1 to 4

1.1. Materials and Methods

Materials. Anhydrous *N*,*N*-dimethylformamide (DMF) was prepared from amine-free DMF (Alfa Aesar) using a Pure Process Technology Solvent Purification System. Lithium chloride and concentrated sulfuric acid (both from Acros), DMSO-d_6 (Cambridge Isotope Laboratories), methyl 3,5-dihydroxybenzoate (**6,** Biosynth), and 2,5-dihydroxybenzoic acid (**8**, Combi-Blocks) were used as received. All other chemicals were purchased from Thermo Fisher Scientific and used as received. Deionized water was used for all aqueous washes and solutions.

General Methods. Unless otherwise stated, all reactions were conducted under a nitrogen atmosphere using anhydrous solvents in glassware that was dried in an oven (120 °C) for at least 30 min and cooled under vacuum prior to use. Room temperature denotes ambient temperature in our laboratories, 23 ± 2 °C.

Column chromatography. Column chromatography was carried out either using a Büchi Pure C-810 Flash Chromatography System using prepacked Büchi EcoFlex Silica columns as the stationary phase or manually using SiliaFlash Irregular Silica Gel (SiliCycle; P60, 40–63 µm, 60 Å). Mobile phase solvents are as indicated. Elution of desired products from the Büchi C-810 was monitored using absorbance at 254 nm. Fractions were visualized by thin-layer chromatography on silica gel plates.

Nuclear magnetic resonance (NMR) spectroscopy. NMR spectra were recorded on a JEOL 400 MHz spectrometer at ambient temperature in the indicated solvent. ^{1}H and ^{13}C spectra were referenced to the signal arising from residual non-deuterated solvent. Data are represented as follows: chemical shift, multiplicity (s = singlet, d = doublet, t = triplet, q = quartet, m = multiplet), coupling constants, integration.

Mass spectrometry. High-resolution mass spectrometry (HRMS) data were recorded on a Thermo Scientific Q Exactive Plus Hybrid Quadrupole-Orbitrap mass spectrometer in electrospray ionization mode. Samples were prepared at a concentration of 10 µg/mL in a 50:50 mixture of MeCN and water + 0.1 % formic acid.

High performance liquid chromotography (HPLC). The purity of dendrons **3** and **4** was determined by analyzing samples on a PerkinElmer LC Column Oven LC 300 HPLC with a PerkinElmer Brownlee column (2.7 µm, C18 SPP, 75 × 3.0 mm). Samples were dissolved in MilliQ water (0.3 mg/mL) and filtered through a centrifugal tube filter to remove particulates. Aliquots (10 µL) were loaded onto the column with an autosampler and eluted at a flow rate of 1 mL/min for 10 min from 0—100% MeCN. Elution was monitored using absorbance at 254 nm.

1.2. Experimental Procedures

Dendrons **1** and **2** were synthesized as described in our previous work.[1] The synthesis of **3** and **4** (**Scheme S1**) follow the same synthetic procedure as **2**, using methyl 3,5-dihydroxybenzoate (**6**) or methyl 2,5-dihydroxybenzoate (**9**), respectively.

Scheme S1. Synthesis of Amphiphilic Dendrons 3 and 4

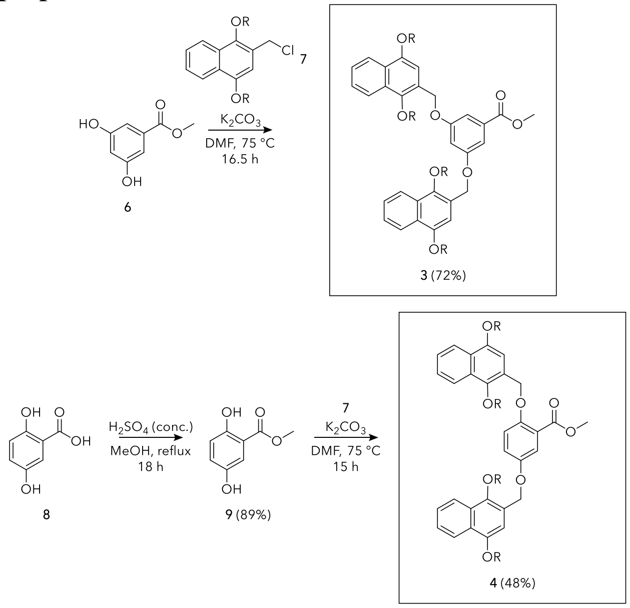

Methyl 3,5-bis((1,4-bis((2,5,8,11-tetraoxatridecan-13-yl)oxy)naphthalen-2-yl)methoxy)benzoate (3).[1] To a mixture of methyl 3,5-dihydroxybenzoate (**6**, 81 mg, 0.48 mmol, 1.0 equiv) and K_2CO_3 (0.40 g, 2.88 mmol, 6.0 equiv) in DMF (5 mL) was added dropwise **7** (0.58 g, 0.98 mmol, 2.05 equiv). The reaction mixture was stirred at 75 °C under N_2 for 16.5 h. The mixture was allowed to cool to room temperature and poured into water (45 mL). The reaction mixture was transferred to a separatory funnel, and the aqueous layer was washed with EtOAc (20 mL × 5). The combined organic layers were washed with LiCl (0.5 M aq., 25 mL × 3), water (20 mL × 2), and NaCl (sat. aq., 20 mL), dried with $MgSO_4$, and concentrated under vacuum. The product was purified by column chromatography (EtOAc to 9:1 EtOAc/MeOH) to give **3** as a pale-yellow oil (0.44 g, 72%; purity by HPLC: ≥97%).

^{1}H NMR (500 MHz, DMSO-*d*$_6$): δ 8.17 (dd, J = 7.8, 1.9 Hz, 4H), 7.63 – 7.53 (m, 4H), 7.26 – 7.23 (m, 2H), 7.10 (q, J = 2.0 Hz, 1H), 7.03 (d, J = 1.3 Hz, 2H), 5.32 (s, 4H), 4.24 (dd, J = 6.1, 3.0 Hz, 4H), 4.12 – 4.07 (m, 4H), 3.89 – 3.85 (m, 4H), 3.84 (d, J = 1.3 Hz, 3H), 3.76 – 3.72 (m, 4H), 3.67 – 3.63 (m, 4H), 3.58 – 3.40 (m, 40H), 3.37 (ddd, J = 5.9, 3.7, 1.4 Hz, 8H), 3.19 (d, J = 1.2 Hz, 6H), 3.18 (d, J = 1.2 Hz, 6H).

^{13}C NMR (126 MHz, DMSO-*d*$_6$): δ 165.91, 159.65, 150.52, 146.22, 131.63, 128.20, 126.88, 126.03, 125.98, 124.80, 122.30, 122.00, 107.93, 105.99, 74.80, 71.25, 71.23, 70.11, 69.97, 69.86, 69.84, 69.80, 69.78, 69.72, 69.65, 69.57, 69.54, 68.96, 68.03, 65.03, 58.05, 58.00, 52.30.

HRMS (ESI/Q-TOF) *m*/*z*: $[M+Na]^+$ calcd for $C_{66}H_{96}O_{24}Na^+$ 1295.6189; found 1295.6191.

Methyl 2,5-dihydroxybenzoate (9).[2] To a solution of **8** (5.0 g, 32.4 mmol, 1.0 equiv) in MeOH (50 mL) was added dropwise H_2SO_4 (conc.) (1.5 mL, 28.3 mmol, 0.9 equiv). The reaction mixture was stirred at reflux for 18 h under ambient atmosphere. The solution was allowed to cool to room temperature and concentrated under vacuum. The residue was diluted in EtOAc (65 mL). $NaHCO_3$ (sat. aq., 50 mL) was added and stirred at room temperature for 15 min. The reaction mixture was transferred to a separatory funnel, and the organic layer was washed with $NaHCO_3$ (sat. aq., 30 mL), water (30 mL) and NaCl (sat. aq., 30 mL), dried with $MgSO_4$, and concentrated under vacuum to give **9** as a white solid (5.47 g, 89%) used in the next step without further purification.

^1H NMR (500 MHz, DMSO-d_6): δ 9.93 (s, 1H), 9.21 (s, 1H), 7.14 (dd, J = 3.2, 1.5 Hz, 1H), 6.97 (ddd, J = 8.9, 3.1, 1.5 Hz, 1H), 6.82 (dd, J = 8.9, 1.5 Hz, 1H), 3.86 (d, J = 1.5 Hz, 3H).

^{13}C NMR (126 MHz, DMSO-d_6): δ 169.34, 153.25, 149.64, 124.10, 124.03, 123.86, 123.80, 118.43, 118.31, 118.29, 118.22, 118.18, 118.12, 118.09, 118.06, 114.32, 114.23, 114.06, 113.97, 112.45, 52.55, 52.52, 52.49, 52.46, 52.43, 52.40, 52.37, 51.77.

Methyl 2,5-bis((1,4-bis((2,5,8,11-tetraoxatridecan-13-yl)oxy)naphthalen-2-yl)methoxy)benzoate (4).[1] To a mixture of methyl 2,5-dihydroxybenzoate (**9**, 0.23 g, 1.38 mmol, 1.0 equiv) and K_2CO_3 (1.14 g, 8.28 mmol, 6.0 equiv) in DMF (25 mL) was added dropwise **7** (1.67 g, 2.83 mmol, 2.05 equiv). The reaction mixture was stirred at 75 °C under N_2 for 15 h. The mixture was allowed to cool to room temperature and poured into water (75 mL). The reaction mixture was transferred to a separatory funnel, and the aqueous layer was washed with EtOAc (45 mL × 5). The combined organic layers were washed with LiCl (0.5 M aq., 45 mL × 3), water (45 mL × 2), and NaCl (sat. aq., 45 mL), dried with $MgSO_4$, and concentrated under vacuum. The product was purified by column chromatography (EtOAc to 9:1 EtOAc/MeOH) to give **4** as a pale-yellow oil (0.84 g, 48%; purity by HPLC: ≥97%).

^1H NMR (500 MHz, DMSO-d_6): δ 8.16 (dt, J = 7.9, 1.8 Hz, 4H), 7.62 – 7.51 (m, 4H), 7.37 (t, J = 1.8 Hz, 1H), 7.26 (d, J = 2.6 Hz, 2H), 7.15 (d, J = 1.3 Hz, 1H), 7.02 (d, J = 1.3 Hz, 1H), 5.29 (d, J = 31.1 Hz, 4H), 4.25 (q, J = 4.6 Hz, 4H), 4.09 (q, J = 4.8 Hz, 4H), 3.91 – 3.84 (m, 4H), 3.81 – 3.74 (m, 7H), 3.68 – 3.44 (m, 42H), 3.40 – 3.35 (m, 8H), 3.19 (dd, J = 4.3, 1.4 Hz, 12H).

^{13}C NMR (126 MHz, DMSO-d_6): δ 151.97, 151.35, 150.48, 150.41, 146.09, 145.56, 128.21, 128.17, 125.90, 125.67, 125.42, 125.14, 121.39, 71.25, 70.11, 70.00, 69.86, 69.78, 69.57, 58.07, 57.99, 57.94.

HRMS (ESI/Q-TOF) *m*/*z*: $[M+Na]^+$ calcd for $C_{66}H_{96}O_{24}Na^+$ 1295.6189; found 1295.6179.

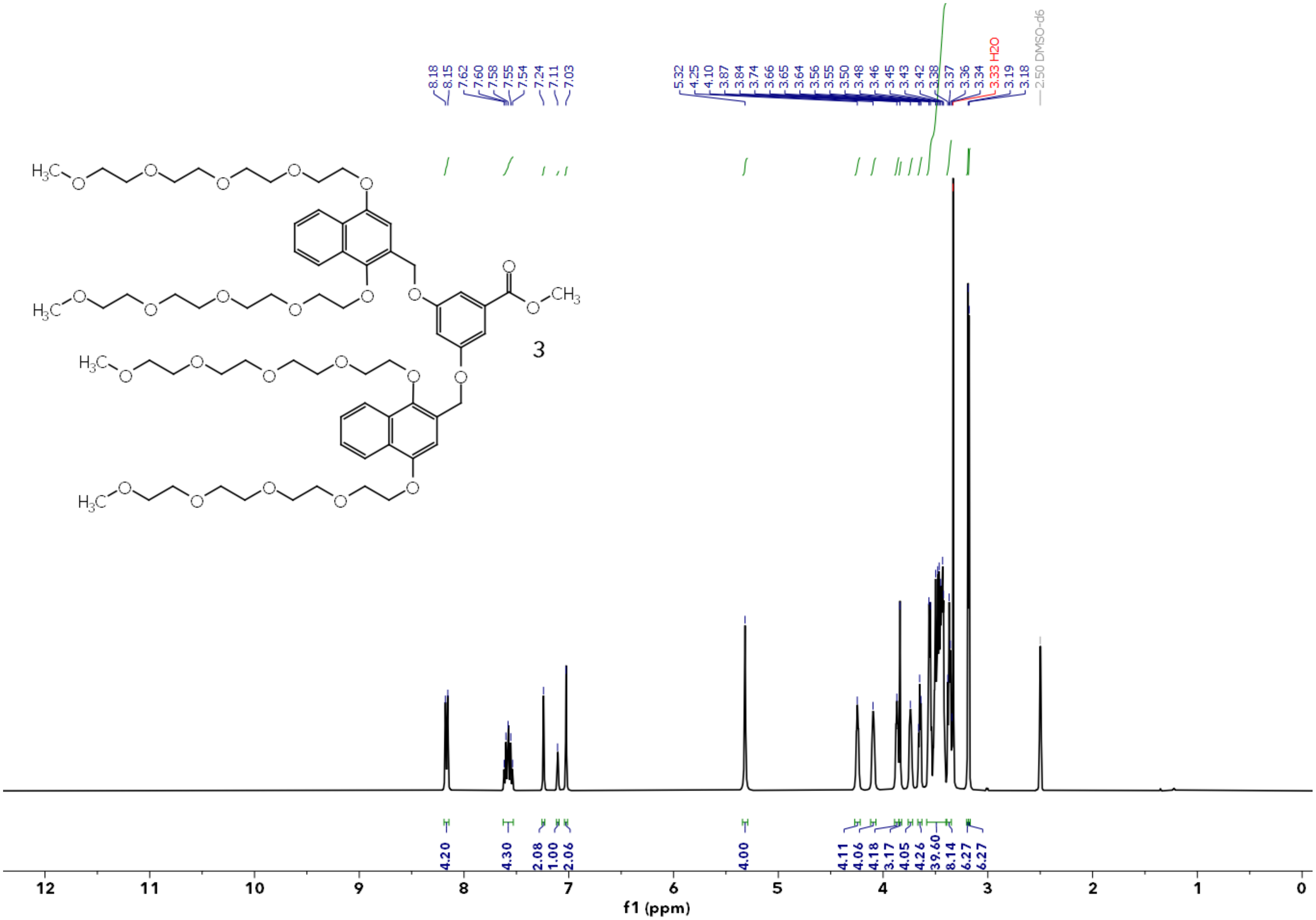

Figure S1. ^{1}H NMR (400 MHz) spectrum of **3** measured in DMSO-d_6 at ambient temperature.

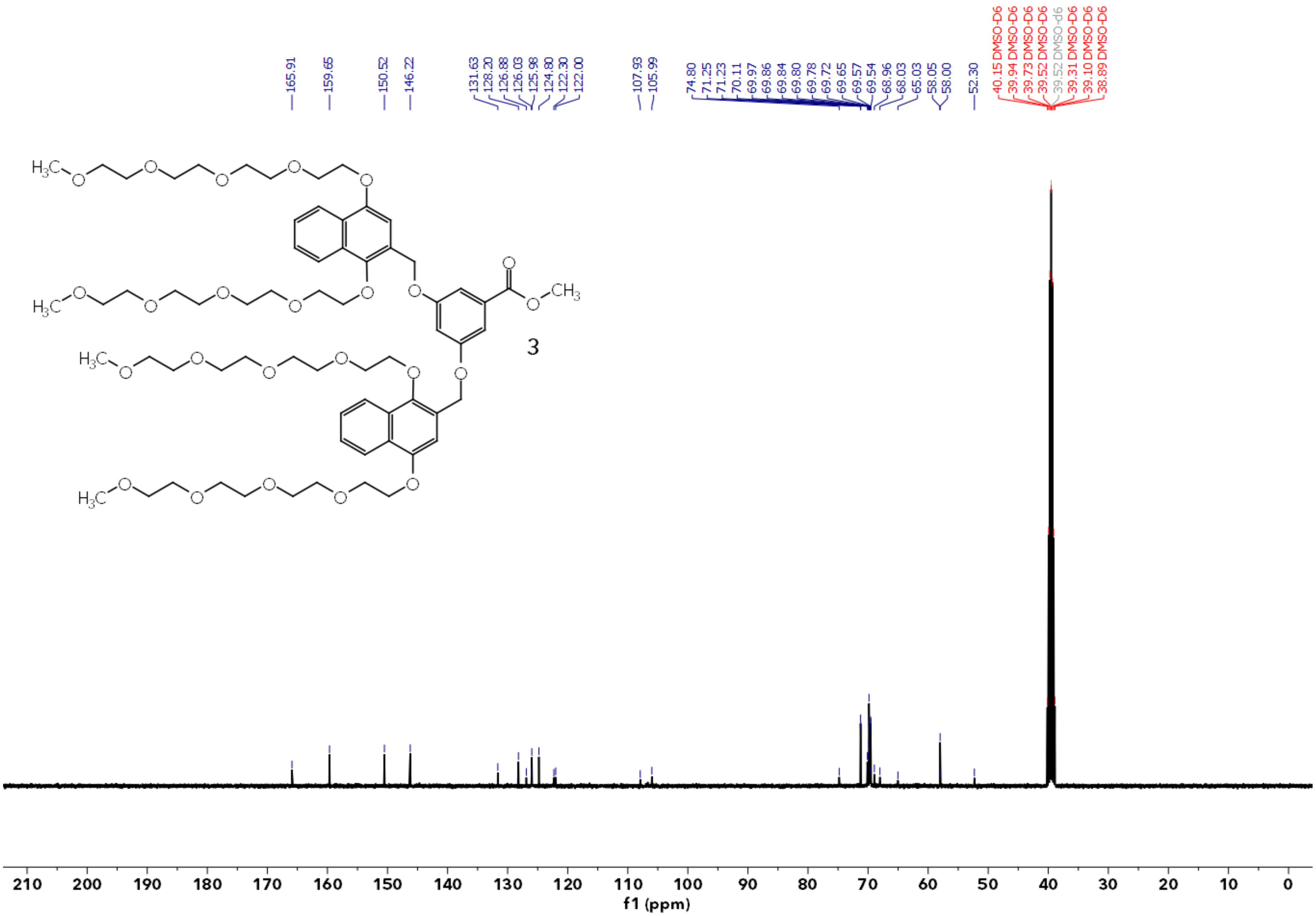

Figure S2. ^{13}C NMR (400 MHz) spectrum of **3** measured in DMSO-d_6 at ambient temperature.

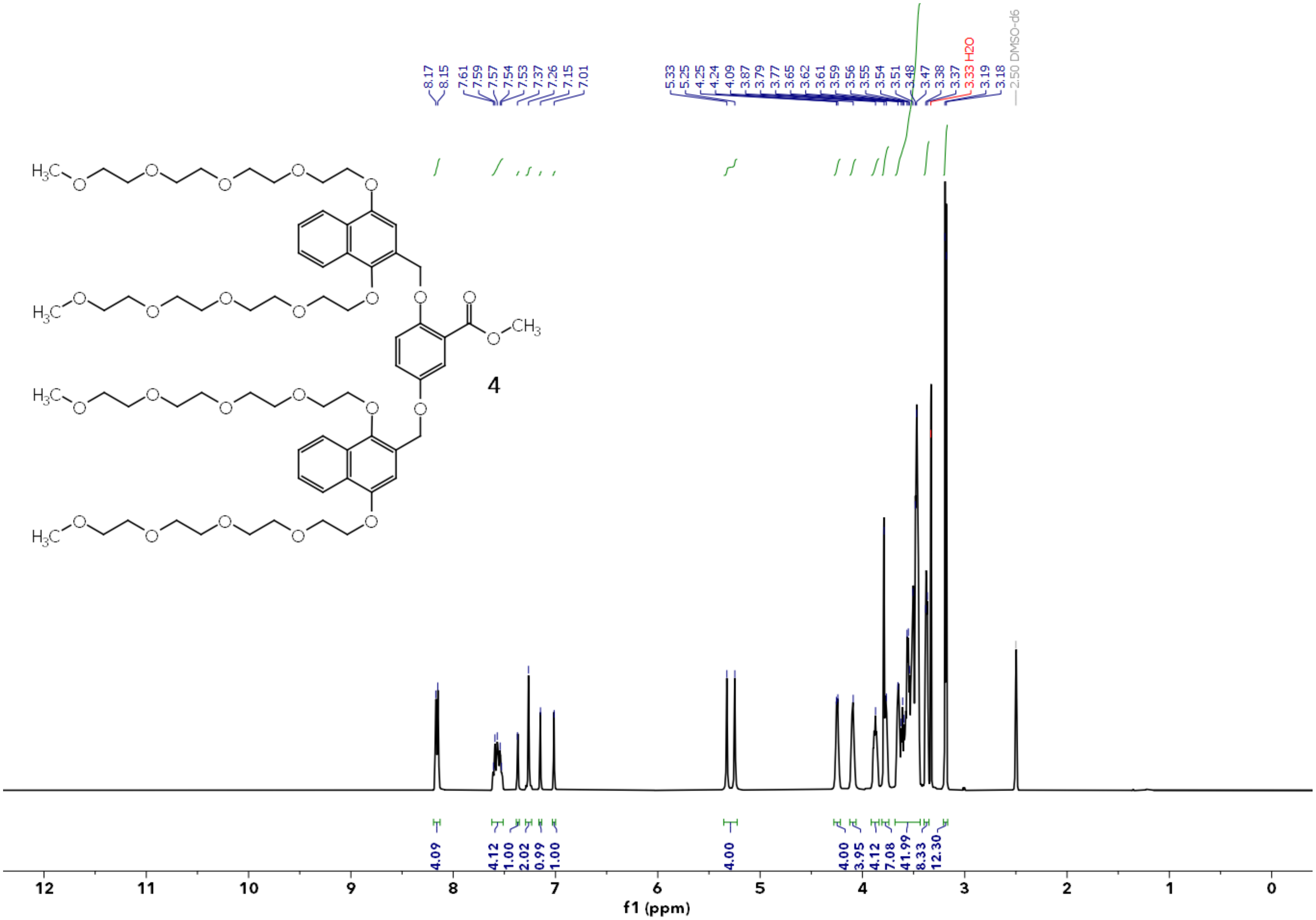

Figure S3. ^{1}H NMR (400 MHz) spectrum of **4** measured in DMSO-d_6 at ambient temperature.

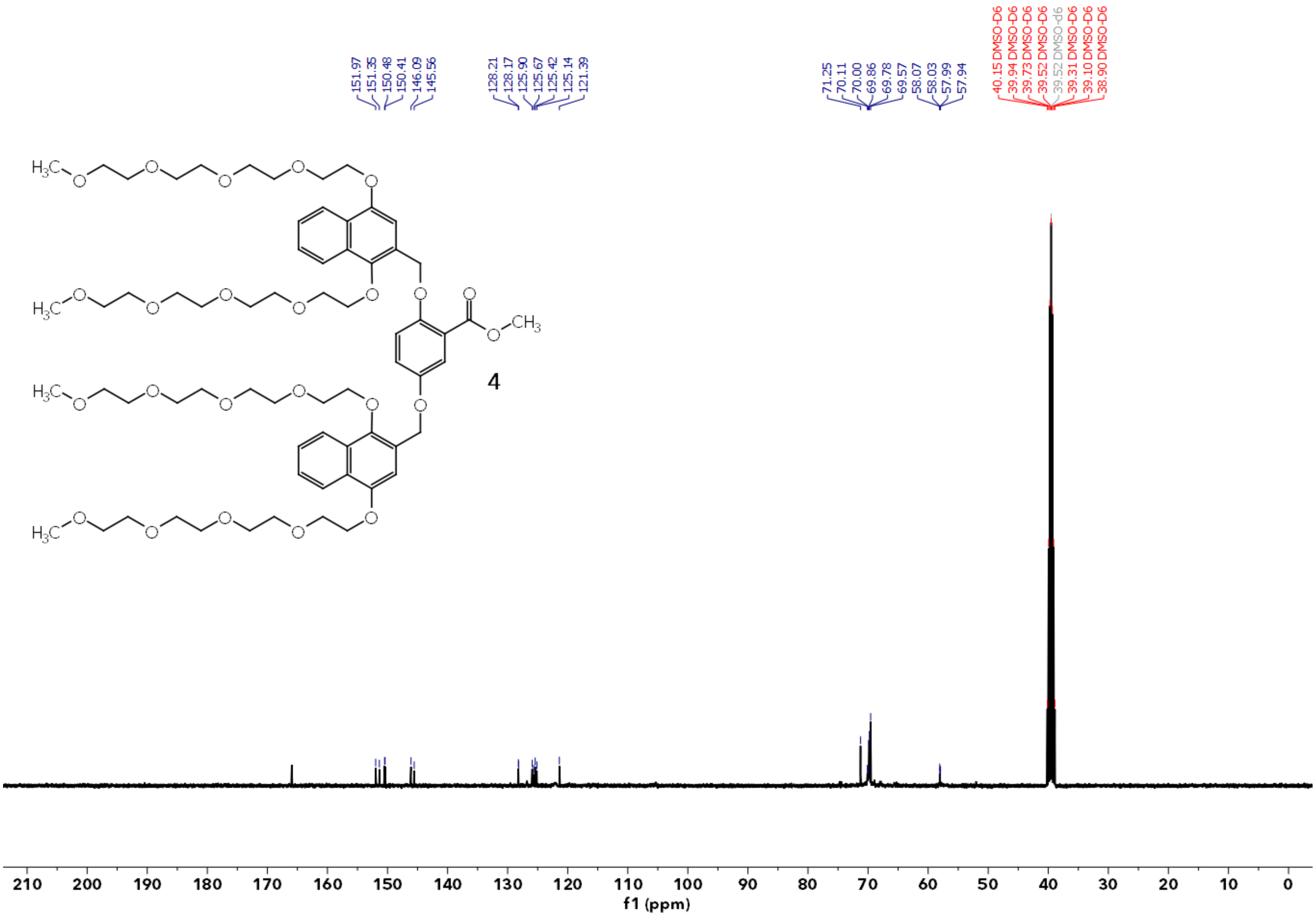

Figure S4. ^{13}C NMR (400 MHz) spectrum of **4** measured in DMSO-d_6 at ambient temperature.

2. Atomic Force Microscopy

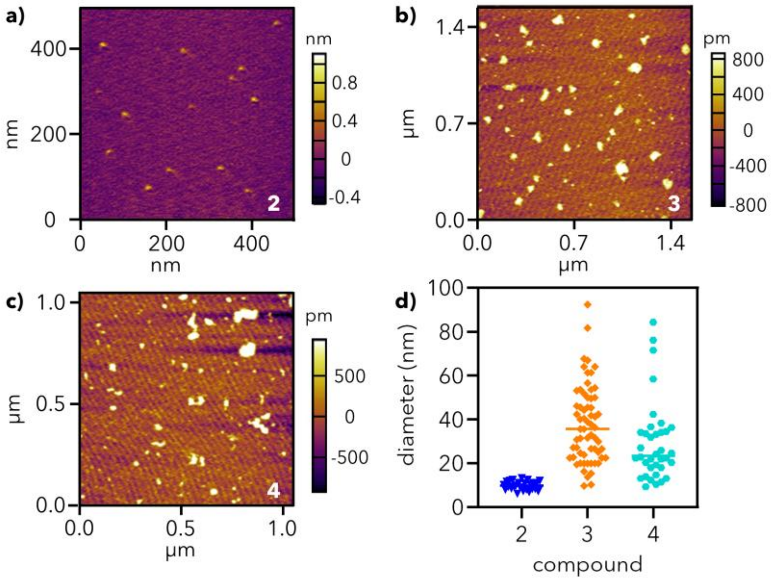

Figure S5. Atomic force microscopy (AFM) images of second generation dendrons **2**, **3**, and **4**. **(a–c)** Representative AFM height images of **(a)** dendron **2**, **(b)** dendron **3**, and **(c)** dendron **4** spin-coated from aqueous solutions (30 µM in MilliQ water) on mica substrates. **(d)** Analysis of the circle equivalent diameter of nanoparticles observed for each dendron. Diameter data were collected for **2** from two images (n = 25), for **3** from two images (n = 63), and for **4** from one image (n = 36). Note that due to the radius of the AFM tip (≤ 7 nm), particles of **2** may not be fully resolved and may be smaller or more irregular than indicated by the data in panel (d).

3. Synthesis and Characterization of F19V Peptide

Ac-KLVVFAE-NH_2 (the F19V mutant of amyloid beta peptide fragment 16–22, hereafter "F19V") was synthesized using Fmoc-protected solid-phase peptide synthesis techniques. Protected amino acids (Aapptec) were used in 4× excess. Amino acids were dissolved in DMF and activated with 4× excess of 1-hydroxybenzotriazole (HOBt) and hexafluorophosphate benzotriazole tetramethyl uronium (HBTU) and ~20× excess of *N,N*-diisopropylethylamine (DIPEA) for 10 min. Activated amino acids were added to deprotected Fmoc-Rink Amide OctaGel Resin (Aapptec, 1% DVB, 0.6 mmol/g) and allowed to couple for 1 h. Fmoc groups were removed, and the resin was deprotected using piperidine in DMF (20% v/v) for 20 min. Following the addition of the final amino acid, the *N*-terminus was acetylated using acetic anhydride in DMF (20% v/v) for 10 min. The peptide was then cleaved from the resin with a solution of 95/2.5/2.5% v/v/v of trifluoroacetic acid (TFA)/triisopropylsilane (TIPS)/H_2O for 1 h, followed by the addition of fresh cleavage cocktail for an additional hour. The resulting peptide was concentrated; ice-cold diethyl ether was added to precipitate the peptide. The peptide was collected via centrifugation and the pellet was dissolved in 60% v/v MeCN/water, frozen and lyophilized.[3]

The peptide was purified via preparatory scale reverse-phase high-performance liquid chromatography (RP-HPLC) on an Interchim Puriflash 4125 instrument with a Phenomenex Gemini column (10 µm, C18 Axia, 250 × 50 mm) with a binary gradient of MeCN and water with 0.1% TFA. The eluent was monitored at 215 and 254 nm for fraction collection. Peptide mass was confirmed using matrix-assisted laser desorption-ionization time-of-flight (MALDI-TOF) mass spectrometry (Shimadzu Axia Performance; **Figure S6a**), and peptide purity was determined using analytical RP-HPLC (Shimadzu LC-2010A equipped with a Phenomenex Gemini 5 µm, C18, 110 Å, 250 × 4.6 mm column). Pure fractions were combined and lyophilized. Peptide concentration was determined by dissolving the lyophilized peptide powder in 60% v/v MeCN/water to prevent premature aggregation, and analyzing via analytical HPLC for comparison to a concentration curve calibrated by amino acid analysis (UC Davis, Davis, CA, USA; **Figure S6b**). Aliquots of the peptide stock solution were separated into low protein binding microcentrifuge tubes, frozen, and lyophilized.

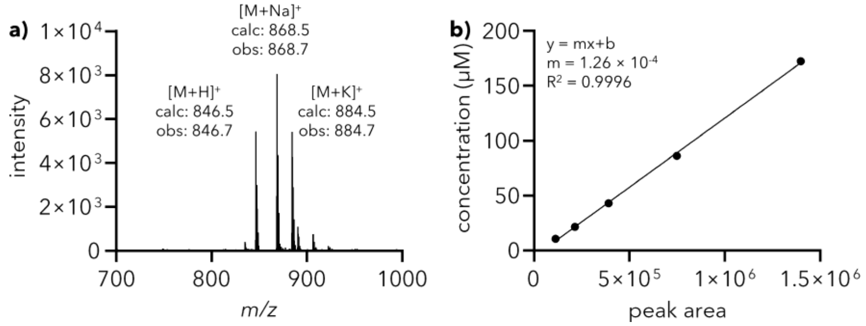

Figure S6. Characterization of F19V. **(a)** MALDI-TOF MS spectra of F19V. α-Cyanohydroxycinnamic acid was used as the matrix and TOF standard mix (Shimadzu) was used as a standard. **(b)** Calibration curve for determination of F19V concentration by HPLC.

4. Supporting Data for Congo Red Peptide Binding Assays

4.1. Data for Individual Replicates

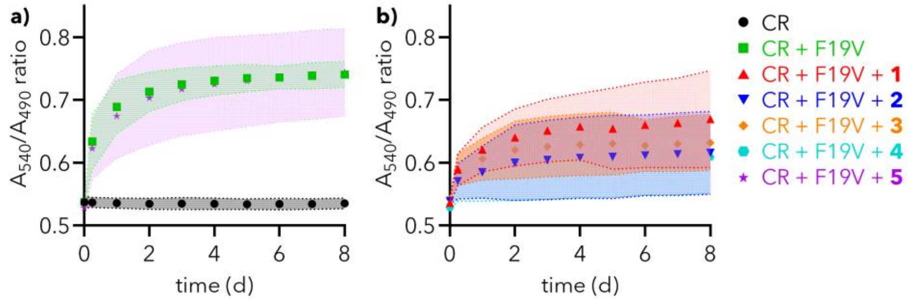

Figure S7. Congo Red (CR) binding assays with F19V. **(a)** A_{540}/A_{490} values for solutions of CR alone (100 µM), CR and F19V (100 µM), and **5** (120 µM). **(b)** A_{540}/A_{490} values for solutions of CR, F19V and either **1**, **2**, **3**, or **4** (30 µM). Error ranges indicate mean ± SD (n = 3).

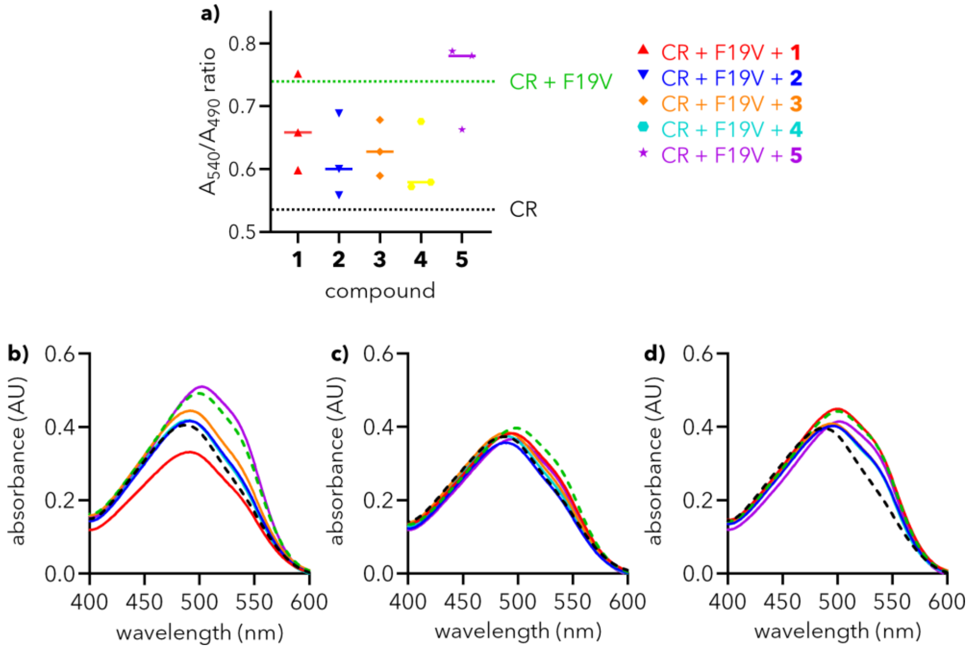

Figure S8. Individual replicates for the CR binding assay. **(a)** A_{540}/A_{490} values for solutions of CR, F19V and either **1**, **2**, **3**, or **4** (30 µM) or **5** (120 µM) at 8 days. Horizontal bars represent mean value (n = 3). Mean values for CR alone (100 µM) and CR and F19V (100 µM) are shown as broken black and green horizontal lines, respectively. **(b–d)** UV-vis spectra of individual scientific replicates at 8 days. Color code is the same as in (a).

4.2. Control Experiments with Congo Red and Dendron

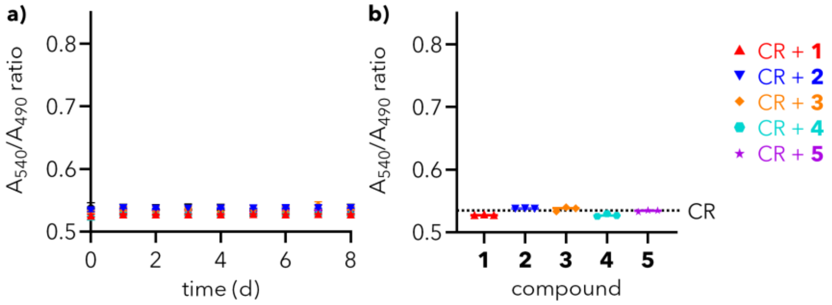

Figure S9. CR and dendron control experiments. **(a)** A_{540}/A_{490} values for solutions of CR with either **1**, **2**, **3**, or **4** (30 μM) or **5** (120 μM) over 8 days. Error bars indicate mean ± SD (n = 3). **(b)** A_{540}/A_{490} values for solutions of CR with either **1**, **2**, **3**, or **4** (30 μM) or **5** (120 μM) at 8 d. CR alone (100 μM) is shown as a broken black horizontal line.

4.3. Additional TEM Images

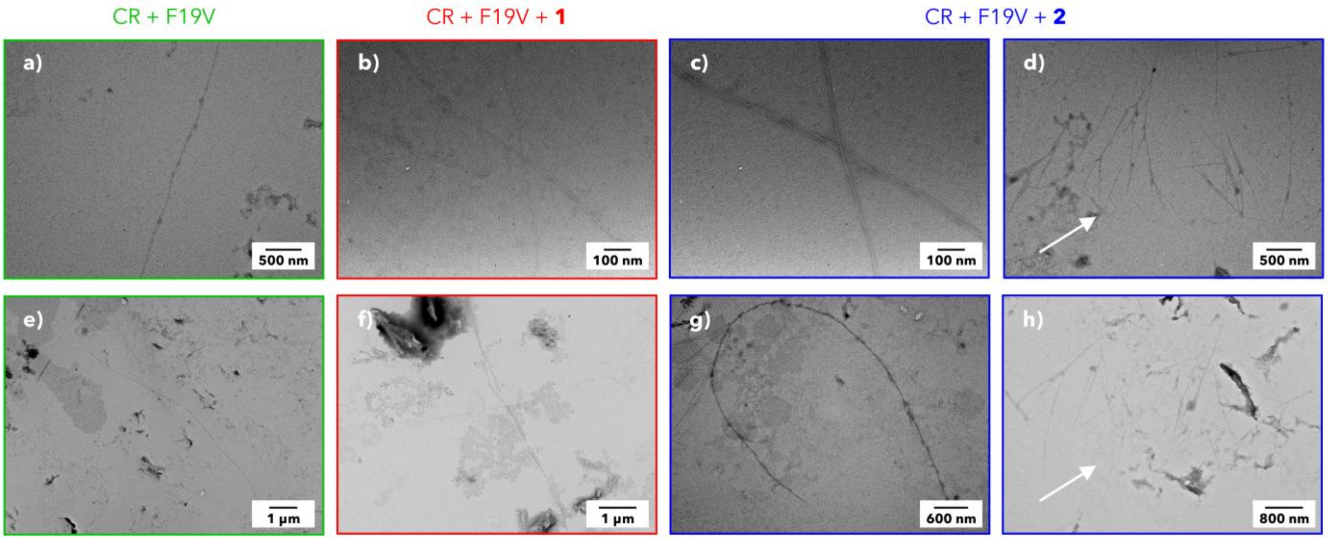

Figure S10. Additional TEM images of CR-bound F19V fibrils. Solutions of CR (100 μM) and F19V (100 μM) **(a, e)** alone and with either **(b, f)** dendron **1** (30 μM) or **(c**, **d**, **g**, **h)** dendron **2** (30 μM). Samples were taken at day 8 directly from the cuvettes from which UV-vis data in **Figure 2** were recorded. White arrows in **d** and **h** highlight shortened fibrils observed in solutions with **2**.

5. Supporting Data for Insulin Aggregation Assays

5.1. Determination of Insulin Oligomerization State by SEC

Size exclusion chromatography (SEC) was performed on a BioRad NGC fast protein liquid chromotography (FPLC) system at 4 °C using a size exclusion column with a 70 kDa exclusion limit (Cytiva Superdex 75 Increase). To determine if commerically available insulin was monomeric or hexameric, an SEC trace of insulin (0.4 mg/mL) was compared with that of gel filtration protein standard (BioRad, catalog # 1511901). The gel filtration standard was diluted according to the manufacturer's directions. For both the gel filtration standard and insulin, 500 µL were injected onto the column at a flow rate of 0.5 mL/min and eluted at the same rate using filtered 1 × PBS (30 mL). Elution was monitored using absorbance at 280 nm.

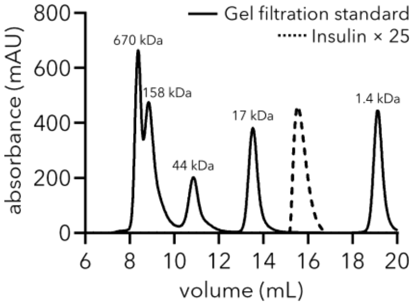

Figure S11. SEC of commercially available insulin. Chromatographs (A_{280}) of gel filtration standard (solid line) and insulin (broken line). The absorbance of insulin was multipled by 25 to better visualize the elution peak. The molecular weights of the gel filtration standard are labeled based on data provided by the supplier.

5.2. Control Experiments with Insulin or DTT and Dendrons

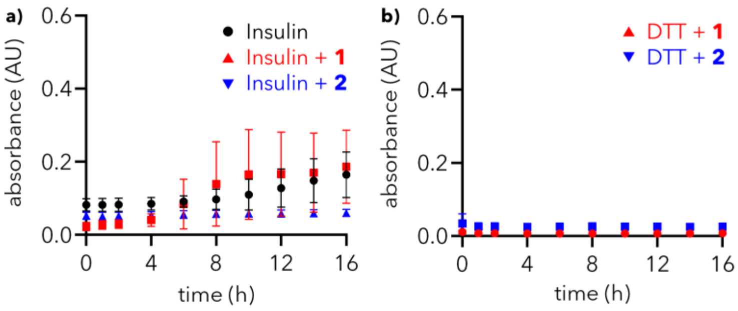

Figure S12. Control experiments for insulin aggregation assay. **(a)** A_{450} values for solutions of insulin (0.4 mg/mL) alone and with either **1** or **2** (250 µM) over 16 h. Data show that dendrons do not induce significant aggregation of insulin. **(b)** A_{450} values for solutions of DTT (100 µM) with either **1** or **2** (250 µM) over 16 h. Data show that dendrons are stable to DTT. Error bars indicate mean ± SD (n = 3).

5.3. Concentration-Dependent Aggregation Inhibition

To determine the minimum concentration of **2** required to prevent insulin aggregation in the presence of DTT, concentration-dependent turbidity assays were performed with dendron concentrations ranging from 50 to 250 µM (**Figure S13a**). Solutions of insulin and DTT exhibit a substantial increase in absorbance over 16 h. Addition of lower concentrations of **2** (50 and 100 µM) show a similar reduction in insulin aggregation to each other, but do not fully prevent aggregation. Concentrations as low as 150 µM show effective insulin stabilization over 16 h, with no significant difference between 150–250 µM (**Figure S13a**). Conversely, as the length of the turbidity assay increases, 150 µM of **2** is not effective in preventing insulin aggregation (**Figure S13b**), instead requiring 250 µM of **2** to maintain efficacy over 5 d.

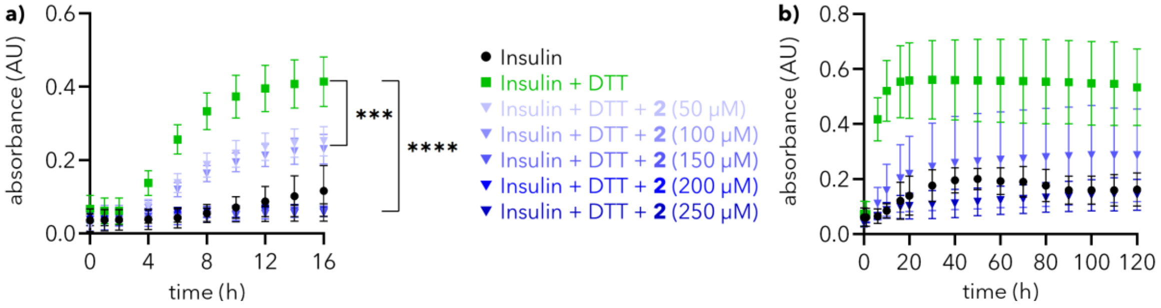

Figure S13. Insulin aggregation assays in reducing conditions with various concentrations of dendron **2**. **(a)** A_{450} values of solutions of insulin (0.4 mg/mL) alone, with DTT (100 µM), and with DTT and **2** (50–250 µM) at 25 °C over 16 h. **(b)** A_{450} values of solutions of insulin (0.4 mg/mL) alone, with DTT (100 µM), and with DTT and **2** (150 and 250 µM) at 25 °C over 5 d. Error bars indicate mean ± SD (n = 4). * denotes $p \leq 0.05$; ** denotes $p \leq 0.01$; *** denotes $p \leq 0.001$; **** denotes $p \leq 0.0001$.

5.4. Additional TEM Images

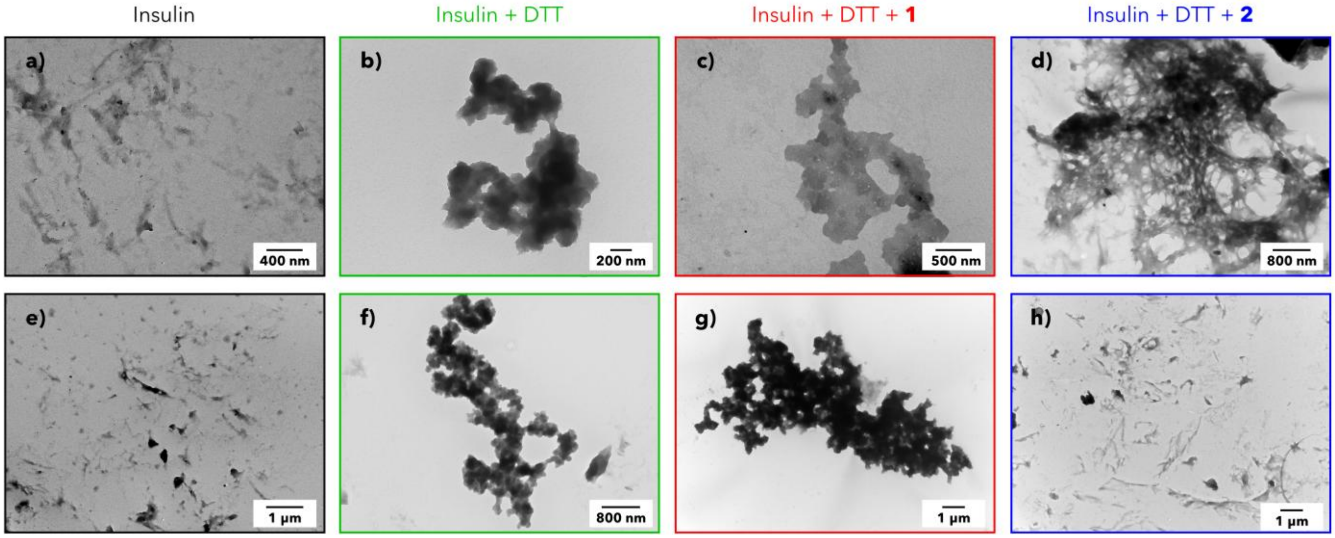

Figure S14. Additional TEM images of insulin aggregation assay. Solutions of insulin (0.4 mg/mL) (**a**, **e)** alone, **(b, f)** with DTT (100 µM) and with either **(c, g)** dendron **1** (250 µM) or (**d**, **h)** dendron **2** (250 µM). Samples were taken after 16 h directly from the 384-well plate from which turbidity data in **Figure 4a** were recorded.

5.5. NMR Study of Dendron Stability with DTT

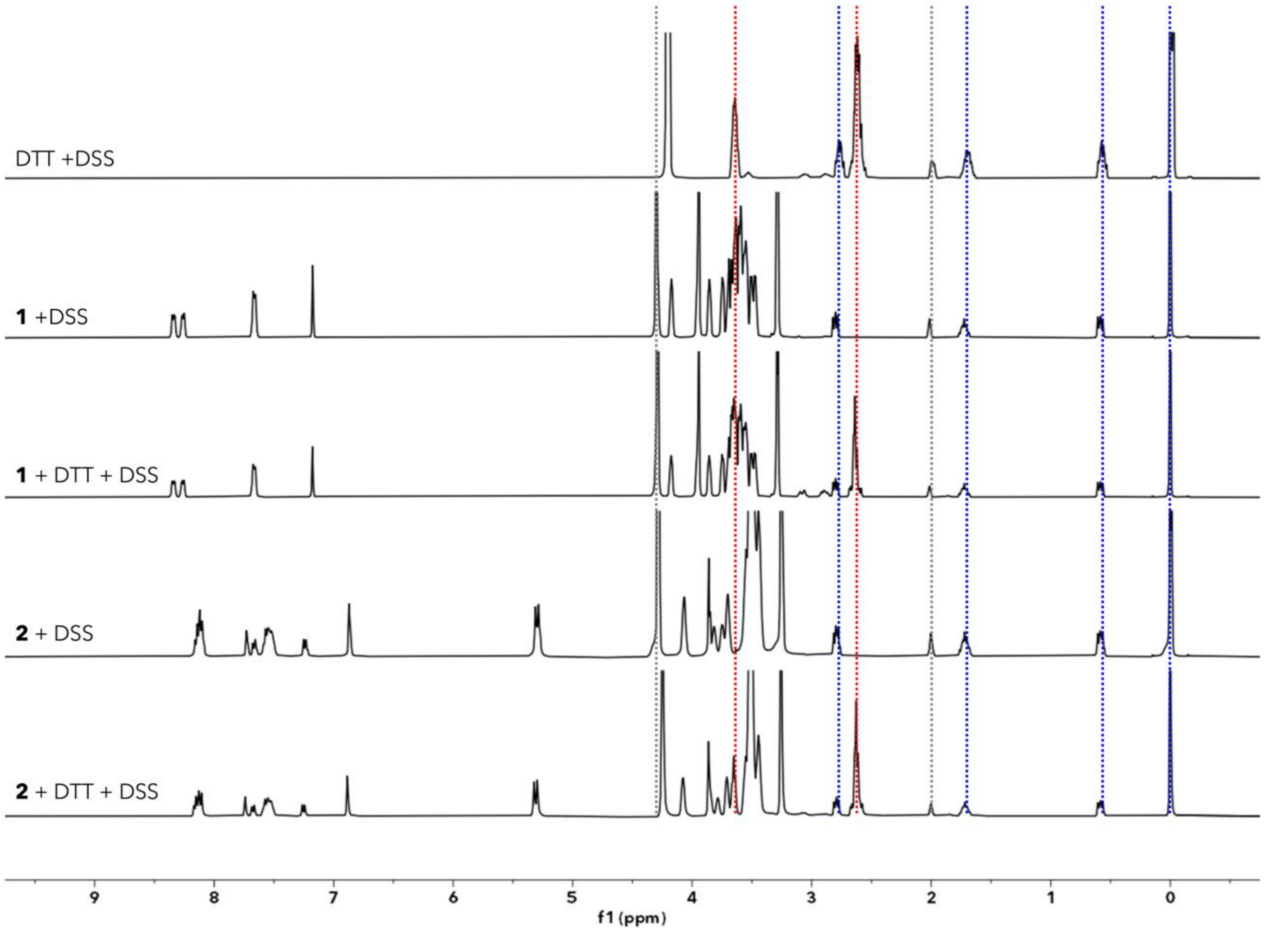

Figure S15. Stability of dendrons **1** and **2** in the presence of DTT. From top to bottom, NMR spectra of: DTT (20 mg/mL, red broken lines); **1** (50 mg/mL); **1** (50 mg/mL) and DTT (20 mg/mL); **2** (50 mg/mL); **2** (50 mg/mL) and DTT (20 mg/mL). All samples were prepared in 1:1 D_2O and MeCN-*d*$_3$ (grey broken lines) with sodium trimethylsilylpropanesulfonate (DSS, TCI America; 10 mg/mL; blue broken lines) as an internal standard to which chemical shifts were referenced. All samples were stored at ambient temperature for 16 h before the spectra were measured. After 16 h, there are no observed changes to the chemical shifts or integrals of dendron peaks, and no additional peaks are present, suggesting the dendrons are stable in the presence of DTT.

5.6. MALDI-TOF MS Study of Insulin Cleavage with DTT

MALDI-TOF mass spectrometry was performed on a Shimadzu Axima Performance in linear mode with a mass range of 1500–15000 g/mol and a power of 100. Horseheart cytochrome C (ProteoMass MALDI-MS standard, Sigma Aldrich) was used as the standard for calibration. Sinapinic acid (Thermo Fisher Scientific) was used as the matrix. Standard solutions were prepared at 10 nmol/mL in MeCN:water:TFA (30:70:1, Shimadzu) and inverted to dissolve. Matrix solutions were prepared at 20 mg/mL in MeCN:water:TFA (50:50:1, Shimadzu), vortexed to dissolve, and centrifuged to clarify.

Insulin aggregation solutions were taken after 16 h directly from the 384-well plate from which turbidity data in **Figure 4a** were recorded. The solutions were desalted using ZipTip C4 resin pipette tips (Millipore) following the manufacturer's instructions except that the pH of the solution after desalting was not adjusted to prevent acid-mediated cleavage of the protein.

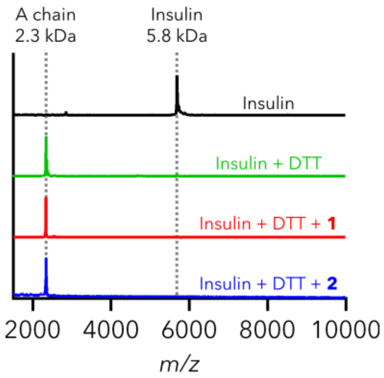

Figure S16. MALDI-TOF mass spectra after insulin aggregation. Normalized spectra of insulin alone, with DTT, and with DTT and either **1** or **2** taken directly from the 384-well plate from which turbidity data in **Figure 4a** were recorded. Sinapinic acid was used as the matrix and horseheart cytochrome C was used as a calibration reference.

6. References for the Supporting Information

1. Piedmont, E. R.; Christensen, E. E.; Krauss, T. D.; Partridge, B. E. Amphiphilic Dendrons as Supramolecular Holdase Chaperones. *RSC Chem. Biol.* **2023**, *4*, 754–759.

2. Percec, V.; Peterca, M.; Sienkowska, M. J.; Ilies, M. A.; Aqad, E.; Smidrkal, J.; Heiney, P. A. Synthesis and Retrostructural Analysis of Libraries of AB_3 and Constitutional Isomeric AB_2 Phenylpropyl Ether-Based Supramolecular Dendrimers. *J. Am. Chem. Soc.* **2006**, *128*, 3324–3334.

3. Jones, C. W.; Morales, C. G.; Eltiste, S. L.; Yanchik-Slade, F. E.; Lee, N. R.; Nilsson, B. L. Capacity for Increased Surface Area in the Hydrophobic Core of B-sheet Peptide Bilayer Nanoribbons. *J. Pept. Sci.* **2021**, *27*, e3334.
